# Novel Model Predicts Type 2 Diabetes Mellitus Patients Complicated With Metabolic Syndrome Using Retrospective Dataset From First Affiliated Hospital of Shenzhen University, China

**DOI:** 10.1155/ije/9558141

**Published:** 2025-04-24

**Authors:** Jinghua Lai, Mingyu Hao, Xiaohong Huang, Shujuan Chen, Dewen Yan, Haiyan Li

**Affiliations:** ^1^Department of Endocrinology, Shenzhen Second People's Hospital, Health Science Center of Shenzhen University, Shenzhen Clinical Research Center for Metabolic Diseases, Shenzhen Center for Diabetes Control and Prevention, The First Affiliated Hospital of Shenzhen University, Shenzhen, China; ^2^Department of Endocrinology, Shenzhen Baoan Shiyan People's Hospital, Shenzhen, China

**Keywords:** clinical prediction model, metabolic syndrome, nomogram, Type 2 diabetes

## Abstract

**Objective:** Metabolic syndrome (MS) is the most important risk factor for Type 2 diabetes mellitus (T2DM) and cardiovascular disease. This study used a retrospective dataset from the First Affiliated Hospital of Shenzhen University and aimed to develop and validate a novel model nomogram based on clinical parameters to predict MS in patients with T2DM.

**Methods:** A total of 2854 patients with T2DM between January 2014 and May 2022 were selected and divided into a training dataset (*n* = 2114) and a validation dataset (*n* = 740). This study used multivariate logistic regression analysis to develop a nomogram for predicting MS in patients with T2DM that included candidates selected in the LASSO regression model. The data were set standardized before LASSO regression. The area under the receiver operating characteristic curve (AUC-ROC) was used to assess discrimination in the prediction model. The calibration curve is used to evaluate the calibration of the calibration nomogram, and the clinical decision curve is used to determine the clinical utility of the calibration diagram. The validation dataset is used to evaluate the performance of predictive models.

**Results:** A total of 2854 patients were eligible for this study. There were 1941 (68.01%) patients with MS. The training dataset included 20 potential risk factors of the patient's demographic, clinical, and laboratory indexes in the LASSO regression analysis. Gender, hypertension, BMI, WC, HbA1c, TG, LDL, and HDL were multivariate models. We obtained a model for estimating MS in patients with T2DM. The AUC-ROC of the training dataset in our model is 0.886, and the 95% CI is 0.871–0.901. Similar to the results obtained from the training dataset, the AUC-ROC of the validation dataset in our model is 0.859, and the 95% CI is 0.831–0.887, thus proving the robustness of the model. The prediction model is as follows: logit (MS) = −9.18209 + 0.14406 ∗ BMI (kg/m^2^) + 0.09218 ∗ WC (cm) + 1.05761 ∗ TG (mmol/L)–3.30013 ∗ HDL (mmol/L). The calibration plots of the predicted probabilities show excellent agreement with the observed MS rates. Decision curve analysis demonstrated that the new nomogram provided significant net benefits in clinical applications.

**Conclusion:** The prediction model of this study covers four clinically easily obtained parameters: BMI, WC, TG, and HDL, and shows a high accuracy rate in the validation dataset. Our predictive model may provide an effective method for large-scale epidemiological studies of T2DM patients with MS and offer a practical tool for the early detection of MS in clinical work.

## 1. Introduction

Diabetes is a huge health problem worldwide and has become one of the most common and severe chronic diseases. According to the data provided by the International Diabetes Federation (IDF), China will have more than 140 million diabetes patients in 2021, accounting for 1/4 of the global diabetes patients, and is the country with the largest number of significant patients in the world, and is expected to reach 174 million by 2045 [1].

Diabetes is a group of metabolic diseases mainly characterized by hyperglycemia, which can present typical symptoms of thirst, polyuria, and loss of weight in clinical practice. When blood glucose rises to a certain extent, acute metabolic disorders such as diabetic ketoacidosis and hyperglycemia hyperosmolar syndrome can be caused, even life-threatening. As a chronic disease, diabetes increases the risk of other diseases through microvascular and macrovascular damage while negatively affecting organ systems such as the eyes, nerves, kidneys, and heart. Chronic hyperglycemia without obvious symptoms can cause microvascular complications such as diabetic kidney disease (DKD), diabetic retinopathy (DR), and diabetic peripheral neuropathy (DPN), as well as macrovascular complications associated with chronic hyperglycemia coronary heart disease, heart failure, stroke, peripheral vascular disease, etc. Studies have shown that various factors are involved in the pathogenesis of diabetes complications, including insulin resistance, hyperglycemia, lipid metabolism disorders, oxidative stress, and inflammation, primarily caused by insulin resistance and hyperglycemia-induced chronic cellular and molecular damage [2, 3]. Economic development, urbanization, aging populations, unhealthy eating habits, sedentary lifestyles, overweight, and obesity are all drivers of the global diabetes epidemic and contribute to the rising prevalence of metabolic syndrome (MS), a group of clinical syndromes characterized by obesity, insulin resistance, diabetes, abnormal lipid metabolism, and hypertension, which significantly increases the risk of cardiovascular diseases and mortality [4–7].

Domestic and foreign studies currently have three definitions of MS: National Cholesterol Education Program Adult Treatment Group III (NCEP-ATP III), IDF, and Chinese Diabetes Society (CDS) [8–10]. Different organizations have slightly different diagnostic criteria for MS, and prevalence estimates vary. Considering that the CDS standard should be more applicable to Chinese people and was updated in 2020, the diagnostic standard of CDS was adopted in this study.

Risk prediction models have considerable potential in the decision-making process of subhealth population and patient management. Risk prediction models can guide screening and interventions to predict the onset of disease. Several risk prediction models have been developed to identify people at high risk for Type 2 diabetes mellitus (T2DM) [11–14]. Studies have shown that early identification of high-risk groups of T2DM, timely lifestyle changes, or drug intervention can delay the process of β-cell failure [15, 16]. Therefore, we looked for a simple and reliable screening tool to identify people at high risk of developing MS in T2DM patients. Early screening, particularly diagnosing T2DM 3 months in advance, can significantly improve blood glucose control and reduce the risk of complications. Through cross-sectional analysis of the clinical characteristics and related influencing factors of hospitalized T2DM patients with MS, this study aims to increase people's attention to MS, timely detection of abnormalities, and early intervention and provide a basis for clinically reducing MS complications and diabetes complications in T2DM patients. Active prevention and treatment of diabetes, effective intervention on risk factors, and effective control of the prevalence rate are significant to our public health endeavors.

## 2. Method

### 2.1. Patients and Study Design

**Figure 1:**
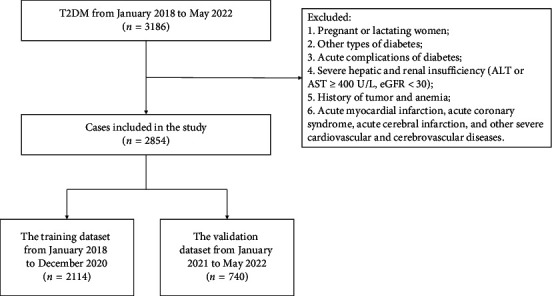
Flowchart of study participants.

This research was a retrospective analysis of patients with T2DM at the First Affiliated Hospital of Shenzhen University between January 2014 and May 2022. The procedure followed in this study aligned with the standards established by the First Affiliated Hospital Ethics Committee of Shenzhen University. The data came from the electronic medical record system, approved by the ethics committee, the ethics approval date for the study is March 29, 2022, and the ethics number is 20220209005. To ensure that our study was adequately powered to detect meaningful effects, we performed a power analysis prior to data collection. Based on previous studies [11, 17], we estimated that a sample size of at least 2000 participants would be required to achieve 80% power with a significance level of 0.05, assuming a moderate effect size. Our final sample size of 2854 patients exceeded this requirement, providing sufficient statistical power to detect clinically relevant associations between the predictors and the presence of MS in patients with T2DM. The inclusion criteria were as follows: T2DM patients. T2DM was diagnosed according to the 2022 American Diabetes Association (ADA) criteria [18], that was, glycated hemoglobin A1c (HbA1c) ≥ 6.5% and/or fasting glucose ≥ 7.0 mmol/L and/or 2-h plasma glucose ≥ 11.1 mmol/L during the oral glucose tolerance test. MS was diagnosed according to the 2020 CDS criteria, that was, to meet the following three or more conditions: (a) abdominal obesity: male waist circumference (WC) ≥ 90 cm and female WC ≥ 85 cm; (b) hyperglycemia: fasting glucose ≥ 6.1 mmol/L, 2-h plasma glucose ≥ 7.8 mmol/L during the oral glucose tolerance test, and/or had been diagnosed with diabetes and treated; (c) hypertension: blood pressure ≥ 130/85 mmHg and/or had been diagnosed with hypertension and treated; (d) triglyceride (TG) ≥ 1.7 mmol/L; and (e) high-density lipoprotein cholesterol (HDL) < 1.04 mmol/L. The exclusion criteria were as follows: (a) pregnant or lactating women; (b) other types of diabetes; (c) acute complications of diabetes; (d) severe hepatic and renal insufficiency (ALT or AST ≥ 400U/L, eGFR < 30); (e) history of tumor and anemia; and (f) acute myocardial infarction, acute coronary syndrome, acute cerebral infarction, and other severe cardiovascular and cerebrovascular diseases. The screening flowchart of the participants in this study was shown in Figure 1. We divided 2854 cases of data into the training dataset (January 2018 to December 2020) and the validation dataset (January 2021 to May 2022), 75% and 25%, respectively. The study was divided into 2 groups by year for external verification. The training dataset was used to establish the model, and the validation dataset was used to evaluate the preliminary performance of the model independently.

### 2.2. Clinical and Laboratory Indexes

The clinical and laboratory indexes were collected, including age, gender, course of diabetes (from diagnosis to hospitalization), smoking, drinking, hypertension, coronary heart disease, diabetic complications, arteriosclerosis (carotid or lower extremity), body mass index (BMI), WC, hip circumference, glycosylated hemoglobin (HbA1c), TG, total cholesterol (TC), low-density lipoprotein cholesterol (LDL), HDL, serum creatinine (Cr), serum uric acid (UA), and 24-h urinary albumin (UAL). The glycosylated hemoglobin was detected by high-pressure liquid chromatography with the Bole D10 glycosylated hemoglobin detector; Hitachi 7600-020 and Olympus 5421 automatic biochemical analyzer were used to detect blood lipids, serum Cr, serum UA, and UAL was determined by routine biochemical colorimetry; Philips epiq-7c color ultrasound was used to detect carotid and lower extremity artery color ultrasound. The laboratory and ultrasonic department provide data, and the instruments and staff are fixed. All results were reviewed during the inspection to ensure the availability and consistency of data. The data of the training dataset and validation dataset were divided into the non-MS group and the MS group according to the diagnostic criteria of MS.

### 2.3. Statistical Analyses

Statistical analyses were performed using R statistical software (Version 4.2.0) and IBM SPSS Statistics (Version 22). Two-tailed *p* values < 0.05 were considered statistically significant. The number of participants with missing data of BMI, WC, hip circumference, HbA1c, TG, TC, LDL, HDL, Cr, UA, and UAL was 8 (0.3%), 66 (2.3%), 66 (2.3%), 42 (1.5%), 33 (1.2%), 33 (1.2%), 34 (1.2%), 33 (1.2%), 14 (0.5%), 23 (0.8%), and 109 (3.8%), respectively. Multiple imputations were used to handle the missing data of covariants. Missing data analysis procedures used missing-at-random (MAR) assumptions [19]. Categorical variables and continuous variables were expressed as frequencies (percentages, %) and means (SDs) or medians (interquartile ranges [IQRs]), respectively. The differences in baseline characteristics between the non-MS and MS groups were assessed using Student's *t* test, the nonparametric Mann–Whitney *U* test for continuous variables and the *χ*^2^ Fisher's exact test for categorical variables.

The Least Absolute Shrinkage and Selection Operator (LASSO) method, suitable for reducing high-dimensional data [20], was used to select the most useful predictive candidates from the training dataset. Candidates with nonzero coefficients were chosen to establish the LASSO model [21]. The data were set standardized before LASSO regression. Multivariate logistic regression analysis to screen for independent clinical predictors related to MS. Each candidate's OR, 95% CI, and *p* value were calculated to predict possible diagnosis. A nomogram was generated based on these risk factors in multivariate analysis. The area under the receiver operating characteristic curve (AUC-ROC) was used to estimate the accuracy and discrimination of the nomogram and these scoring systems in the training and validation datasets. The nomogram provided a quantitative tool to predict the risk of T2DM with MS [22].

Calibration was an agreement between observed outcome frequencies and predicted probabilities. Calibration was studied from graphical representations of the relationship between the observed outcome frequencies and the predicted probabilities (calibration curves). A sensible calibration measure was a likelihood ratio statistic testing the null hypothesis that intercept = 0 and slope = 1. The statistic had a *χ*^2^ distribution with two degrees of freedom (unreliability U-statistic) [23]. We also evaluated average (E-aver) and maximal errors (E-max) between predictions and observations obtained from a calibration curve. The calibration curve was plotted to assess the calibration of the nomogram, and a nonsignificant test statistic implied that the model was calibrated perfectly [24]. Decision curve analysis (DCA) was used to evaluate the clinical value of the predictive model. DCA was conducted to determine the clinical usefulness of the nomogram by quantifying the net benefits at different threshold probabilities in the validation dataset [22, 25].

## 3. Results

### 3.1. General Information on Patients

**Figure 2:**
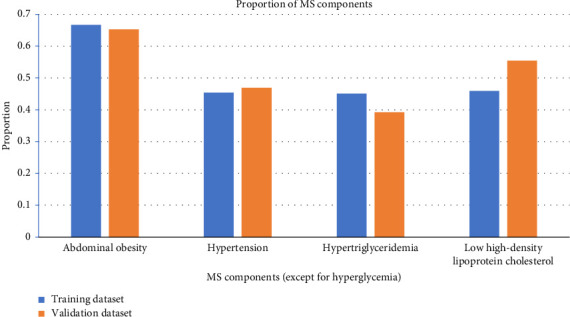
Rate of MS components. The *x*-axis represents the MS components except for path glycemia. The *y*-axis represents the rate. The blue bars were the training dataset, and the orange bars were the validation dataset. The rate of abdominal obesity, hypertension, hypertriglyceridemia, and low high-density cholesterol was compared between the two datasets.

A total of 3186 patients with T2DM were initially enrolled in this study between January 2018 and May 2022. Of them, 2854 patients were eligible for this study. The median age of these eligible patients was 57 years (IQR 48–65). Among them, 1936 (67.84%) patients were male and 1323 (46.36%) had a disease course for over 10 years. The median BMI was 24.6 (IQR 22.6–26.9) kg/m^2^, and the median HbA1c was 8.5% (IQR 7.0%–10.4%). There were 1941 (68.01%) patients with MS. The patients were assigned to two study groups: the training dataset (January 2018 to December 2020) and the validation dataset (January 2021 to May 2022). The training and validation datasets had 2114 and 740 T2DM patients, respectively. There were 1431 (67.69%) patients with MS in the training dataset, and the proportions of abdominal obesity, hypertension, hypertriglyceridemia, and low HDL in MS components were 66.65%, 45.41%, 45.03%, and 45.93%, respectively. There were 510 (68.92%) patients with MS in the validation dataset, and the proportions of abdominal obesity, hypertension, hypertriglyceridemia, and low HDL in MS components were 65.27%, 46.89%, 39.19%, and 55.41%, respectively. The baseline characteristics of the two groups were similar, as shown in Table 1, and the comparison of MS components between the two groups was shown in Figure 2.

### 3.2. LASSO Regression in the Training Dataset

**Figure 3:**
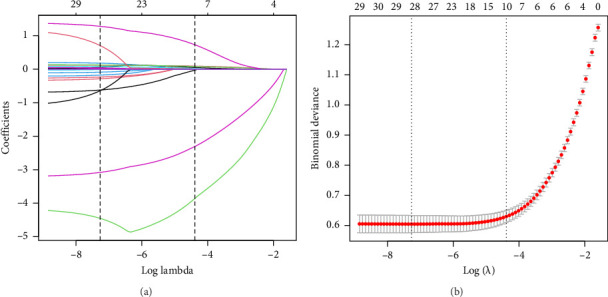
Demographic and clinical feature selection using the LASSO binary logistic regression model. (a) Optimal candidate (lambda) selection in the LASSO model used 10-fold cross-validation via minimum criteria. The area under the receiver operating characteristic curve was plotted versus log (lambda). Vertical lines were drawn at the optimal values using the minimum criteria and the 1 S.E. of the minimum standards; (b) LASSO coefficient profiles of the 20 candidates. A coefficient profile plot was produced against the log (lambda) sequence. A vertical line was drawn at the value selected using 10-fold cross-validation, where optimal lambda resulted in 8 candidates with nonzero coefficients (lambda = 0.0125).

The training dataset included 20 potential risk factors of the patient's demographic, clinical, and laboratory indexes in the LASSO regression analysis, including age, gender, course of diabetes (from diagnosis to hospitalization), smoking, drinking, hypertension, coronary heart disease, diabetic complications, arteriosclerosis (carotid or lower extremity), BMI, WC, hip circumference, HbA1c, TG, TC, LDL, HDL, Cr, UA, and UAL (Figure 3). The variables with nonzero coefficients in the LASSO regression model were considered related to MS. They were selected for further research, including gender, hypertension, BMI, WC, HbA1c, TG, LDL, and HDL. The value of Lambda that the minimum mean cross-validated error.

### 3.3. Development of an Individualized Prediction Model

**Figure 4:**
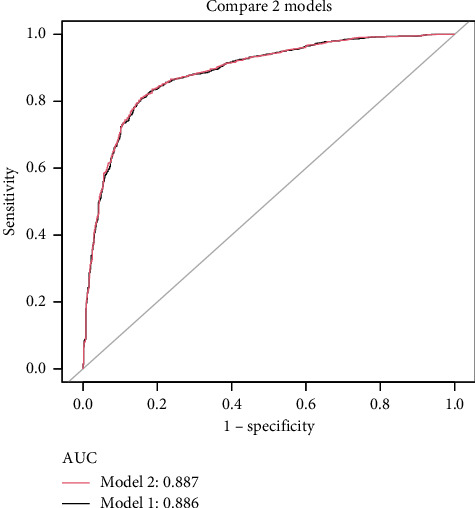
The ROC curves of Model 1 (black line) and Model 2 (red line).

Using the predictors screened by the LASSO regression as independent variables, we constructed two multivariate logistic regression models. These two models' areas under the AUC-ROC were relatively close. The AUC-ROC values, respectively, were 0.886 and 0.887 for these two models (Figure 4). The analysis results of the two models were compared in Table 2. Given that Model 1 incorporated fewer risk factors than Model 2 and the AIC value was minimal and could predict MS in patients with T2DM relatively well, we choose Model 1 as the final risk prediction model for MS in patients with T2DM. The multivariate logistic regression analysis demonstrated that BMI, WC, TG, and HDL were independently associated with MS in T2DM patients (Table 3). Specifically, higher BMI (OR = 1.155, 95% CI: 1.108–1.204, *p* < 0.001), WC (OR = 1.097, 95% CI: 1.080–1.113, *p* < 0.001) and TG (OR = 2.880, 95% CI: 2.409–3.442, *p* < 0.001) were associated with an increased risk of MS, while higher HDL levels (OR = 0.037, 95% CI: 0.021–0.064, *p* < 0.001) were associated with a reduced risk. The results of multivariate logistic regression analyses were shown in Table 3.

**Figure 5:**
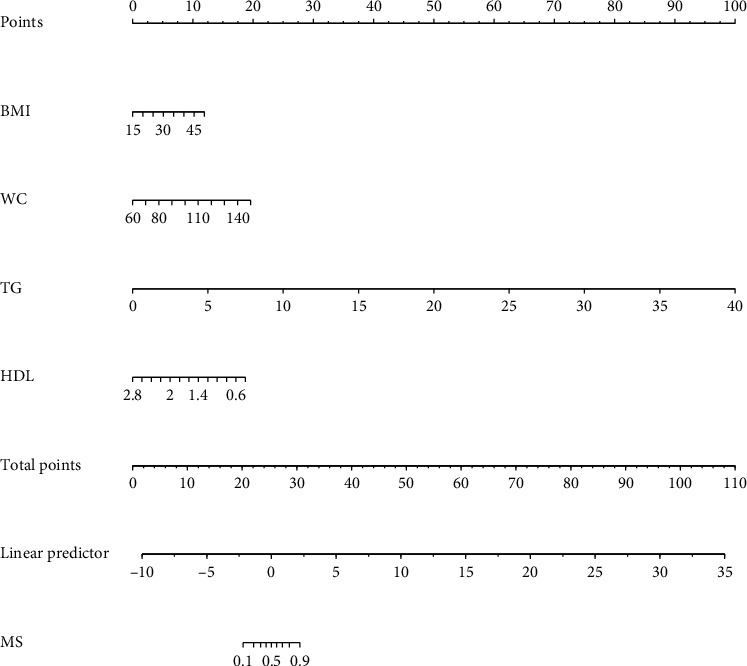
Nomogram predicting MS in T2DM. The nomogram was developed in the training dataset with BMI, WC, TG, and HDL. Points of each variable were acquired by drawing a straight line upward from the corresponding value to the “points” line. Then, sum the points received from each variable and locate the number on the “total points” axis. To conclude the patient's probability of having MS in T2DM, draw a straight line to the corresponding “probability of MS in T2DM” axis. Units: BMI, kg/m^2^; WC, cm; TG, mmol/L; HDL, mmol/L.

**Figure 6:**
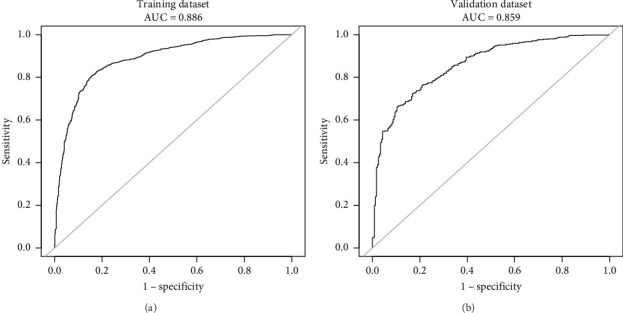
The ROC curves of the novel model in the training dataset (a) and validation dataset (b).

This research developed a model incorporating the four potential predictors and presented it as a nomogram (Figure 5). The nomogram was assigned a specific score, and the total score was used to obtain the probability of predicting MS. The ratios of the calculated beta were used to evaluate the proportional predictive effects of these variables. The projections from total points on the scales below indicated the estimated probability of MS. Therefore, the best prediction model we proposed was as follows: logit (MS) = −9.18209 + 0.14406 ∗ BMI (kg/m^2^) + 0.09218 ∗ WC (cm) + 1.05761 ∗ TG (mmol/L)–3.30013 ∗ HDL (mmol/L). In the training dataset, the AUC-ROC in our model is 0.886 (95% CI, 0.871–0.901) (Figure 6(a)). The validation dataset was applied to the regression model for performance evaluation. The AUC-ROC is 0.859 (95% CI, 0.831–0.887) (Figure 6(b)). Other analysis results were shown in Table 4.

### 3.4. Apparent Performance of the Nomogram

**Figure 7:**
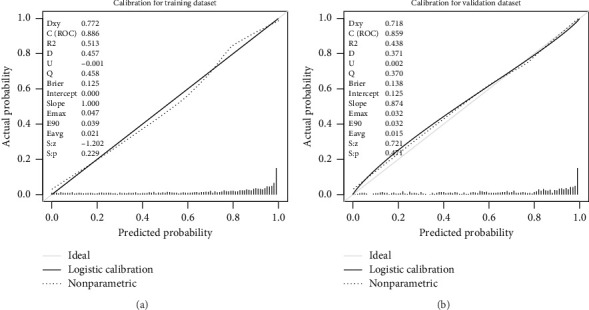
Calibration curve of the novel model in the training dataset (a) and the validation dataset (b). The *x*-axis represented the predicted probability of MS in T2DM. The *y*-axis represented the actual diagnosed MS in T2DM. The diagonal dotted line represented a perfect prediction by an ideal model. The solid line represented the performance of the nomogram, of which a closer fit to the diagonal dotted line means a better prognosis.

The calibration curve of the nomogram for the probability of MS demonstrated good agreement between prediction and observation in the training dataset (Figure 7). To provide a more rigorous assessment of the model's calibration, we calculated the Hosmer–Lemeshow test statistic. The Hosmer–Lemeshow test yielded a *χ*^2^ statistic of 52.45 (*p* < 0.001) in the training dataset and 19.66 (*p* < 0.05) in the validation dataset.

### 3.5. Clinical Use

**Figure 8:**
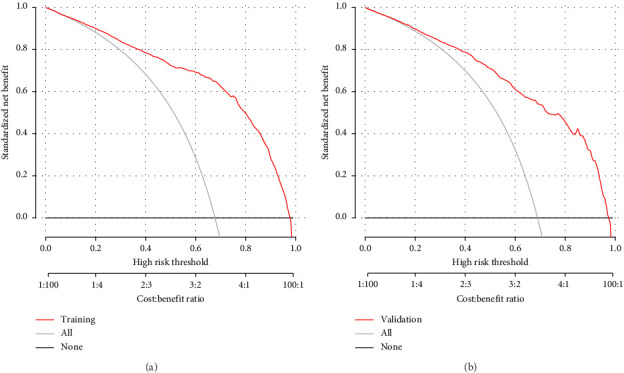
The decision curve analysis of the novel model in the training dataset (a) and validation dataset (b). The black line represented the net benefit when none of the participants was considered to have MS in T2DM. In contrast, the light gray line represented the net benefit when all participants were deemed to have MS in T2DM. The area between the “no treatment line” (black line) and “all treatment line” (light gray line) in the model curve indicated the clinical utility of the model. The farther the model curve was from the black and light gray stripes, the better the clinical use of the nomogram.

The DCA demonstrated that the nomogram provided a net benefit across a wide range of threshold probabilities (Figure 8). While the net benefit at a threshold probability of > 1% was highlighted, this corresponds to an implausibly low risk threshold for MS in T2DM patients. In clinical practice, a more reasonable threshold range for initiating interventions might be between 20% and 50%, where the net benefit of the nomogram remained significantly higher than the “treat all” and “treat none” strategies. For example, at a threshold probability of 30%, the net benefit of the nomogram was 0.45, compared to 0.35 for the “treat all” strategy and 0.10 for the “treat none” strategy. This indicated that the nomogram has significant clinical utility and can help clinicians make more informed decisions about the management of T2DM patients with MS. In the training dataset, the decision curve showed that if the threshold probability of a patient was > 1%, the net benefit was more than 90%. The above results show a broad spectrum of alternative threshold probability in the model, suggesting that the model was a good assessment tool. Therefore, we can use the nomogram to predict the diagnosis of MS.

## 4. Discussion

The core advantage of our model lies in its ability to detect T2DM at an early stage, even though the indicators used (BMI, WC, TG, and HDL) are not novel in themselves. The model achieves this by integrating multiple easily obtainable clinical parameters into a robust predictive framework, which allows for the identification of high-risk individuals before the onset of severe complications. This early detection is crucial because it enables timely interventions, such as lifestyle modifications or pharmacological treatments, which can significantly improve glycemic control and reduce the risk of cardiovascular diseases and other diabetes-related complications [13, 18]. The strength of our model is further supported by its high AUC values in both the training (0.886) and validation (0.859) datasets, demonstrating its reliability and potential for widespread clinical application. Additionally, the simplicity and practicality of the model make it suitable for use in primary care settings, where early screening and intervention can have the most significant impact on patient outcomes [11]. By focusing on easily measurable clinical parameters, our model provides a cost-effective and accessible tool for the early detection of T2DM, particularly in resource-limited settings [1].

Studies have shown that more than 90% of T2DM patients are accompanied by overweight or obesity, and more than 20% of obese patients have diabetes [26, 27]. Each component of MS is an independent risk factor for atherosclerotic cardiovascular disease (ASCVD), the leading cause of death in diabetic patients in China. A combination of these risk factors is directly involved in and contributes to the developing ASCVD, including microvascular dysfunction, coronary atherosclerosis and calcification, heart dysfunction, myocardial infarction, and heart failure. While progress has been made in understanding the cause and outcome of MS, the underlying pathophysiological mechanisms remain unclear, and it is unclear how these concurrent risk factors work together to produce various obesity-related adverse cardiovascular effects [28]. MS alone does not predict the risk of cardiovascular disease worldwide. Still, abdominal obesity—the most common manifestation of MS—is a marker of “adipose tissue dysfunction” critical in clinical diagnosis. In this study, two-thirds of T2DM patients had abdominal obesity, and BMI, WC, and hip circumference in the MS group were significantly higher than those in the non-MS group. In the LASSO regression combined with multivariate logistic regression analysis, BMI and WC were risk factors for T2DM with MS. BMI and WC have been accepted indicators for assessing obesity. The study by Ramirez-Manent et al. pointed out that WC is a core element in detecting insulin resistance, and monitoring WC can see MS early [29]. A cross-sectional study by Yang et al. found that the combined application of BMI and WC was superior to their independent results in identifying MS prevalence in middle-aged and older adults, independent of age, sex, and race [30].

Although insulin resistance has been removed from the clinical definition of MS, most studies believe that insulin resistance is one of the crucial mechanisms of MS and a core mechanism of T2DM, as components of MS, obesity, and dyslipidemia are closely related to insulin resistance. Changes in plasma free fatty acid (FFA) levels and their metabolism are both a cause and a consequence of insulin resistance and T2DM [31–33]. The increase in plasma FFA leads to insulin resistance, further increasing plasma FFA concentration through increased lipolysis, constituting a vicious cycle affecting FFA in T2DM patients. Due to the dysregulation of adipose cells, excess FFA is released into the blood circulation and absorbed by other organs, such as the liver, pancreas, heart, and skeletal muscle, inducing lipotoxicity and affecting cell function [34]. Dyslipidemia is very common in T2DM patients, with a prevalence of 72%–85% [35]. Previous studies have shown that elevated TG and decreased HDL are the characteristic manifestations of diabetic dyslipidemia [35, 36]. Insulin resistance slows the clearance of TG-rich lipoproteins in plasma, leading to hypertriglyceridemia [37]. In hypertriglyceridemia microenvironment, the increased activity of cholesterol ester transfer protein triggers the exchange of TG and cholesterol between lipoprotein particles, thereby increasing the content of TG in HDL [38]. However, due to the structural instability of TG-rich HDL particles and hydrolysis of liver lipase, circulating HDL levels are reduced. A study in China showed that the prevalence of dyslipidemia in rural and urban adults over 40 years old in Northeast China was 60.7% and 66.4%, respectively [39]. The majority of dyslipidemia in the training set of this study was 64.24%, which was similar to the results of the above research; one of the crucial reasons may be the relatively older age of the participants. Therefore, more attention must be paid to detecting and managing dyslipidemia in middle-aged and older adults.

Excessive salt intake is an essential variable of hypertension [40]. A cross-sectional study of 102,216 adults from 18 countries has shown that excessive salt intake is closely related to hypertension [41], and interventional studies have confirmed that salt intake is closely associated with blood pressure [42, 43]. In addition, an observational study in Japan over a follow-up period of about 3 years showed that higher salt intake and gradually increasing salt were associated with increased future blood pressure and hypertension incidence [44]. Hypertension is one of the characteristics of MS, and MS is also ubiquitous in hypertensive patients [45]. Metabolic diseases can amplify hypertension with heart and kidney damage. A meta-analysis showed that MS was associated with an increased risk of major cardiovascular adverse events (MACE), stroke, and cardiovascular death in hypertensive patients. Compared with patients without MS, hypertensive patients with MS had a 55%, 46%, and 45% increased risk of MACE, stroke, and cardiovascular death, respectively [46]. In the Global Cardiometabolic Risk Profile study of patients with hypertensive disease, less than one-third of hypertensive participants had blood pressure values within acceptable ranges. The prevalence of MS was significantly higher in patients with uncontrolled blood pressure compared to patients with well-controlled blood pressure, with 95.3% of patients with both MS and T2DM having unchecked blood pressure [47]. Excessive salt intake activates the sympathetic nerve and renin–angiotensin–aldosterone system (RAAS) [48]. Evidence suggests that tissue activation of the RAAS is involved in endothelial dysfunction and insulin resistance. Hyperinsulinemia may contribute to elevated blood pressure through endothelial dysfunction, proinflammatory cytokines, and increased sympathetic tone [49]. In this study, nearly half of T2DM patients were complicated with hypertension and about 60% were diagnosed with MS. However, due to the retrospective study, there was a lack of data on patients' diet, standard blood pressure control, and use of antihypertensive drugs, etc., so it is impossible to know the influencing factors of hypertension in this population, which is also one of the directions of our future research efforts.

HbA1c can reflect the average blood glucose level in the last 2–3 months and has good stability. Therefore, we opted for HbA1c as the independent variable instead of fasting glucose in LASSO regression. Diabetes self-management and glycemic control among T2DM patients in China are not optimal. A compass study across 10 provinces and cities in China has reported that fewer than 40% of T2DM patients achieved the target HbA1c value of 7% [50]. In the training dataset of this study, only 25.59% of T2DM patients met the standard of glycemic control (HbA1c ≤ 7%). This rate was lower than that reported in similar domestic studies [51–53], and it cannot be ruled out that it might be related to the diabetes knowledge, health literacy, problem-solving ability, and economic conditions of the enrolled patients. Physical activity is a crucial aspect of diabetes self-management vital for maintaining glycemic control in diabetes. Evidence suggests that low physical activity levels are associated with a higher prevalence of MS in T2DM patients [54]. This strongly emphasizes that physical activity is essential not only for diabetes management but also for preventing MS. Over the years, various methods have been explored for glucose control through dietary interventions to prevent MS. These methods include energy restriction, low glycemic index (GI) diets, the Mediterranean diet, and whole grain diets [55]. With the popularity of MS, finding an ease to adhere to and effective diet strategy is still an unresolved problem, and introducing personalized nutrition patterns will be a more significant challenge.

Perimenopausal T2DM patients are mostly accompanied by hypertension, hyperlipidemia, overweight, obesity, etc. They are high-risk groups for cardiovascular diseases [56], which are also components of MS. Estradiol can promote liver uptake and metabolism of LDL, inhibit liver lipase decomposition of HDL, increase HDL content, increase apolipoprotein synthesis, inhibit platelet aggregation, and change the collagen and elastin components on the arterial wall [57, 58]. Therefore, estradiol can protect vascular endothelial cells, inhibit platelet aggregation, regulate blood lipids, and inhibit insulin resistance [59]. Lower estradiol levels in perimenopausal women will increase the risk of centripetal fat accumulation and dyslipidemia, promoting postmenopausal T2DM patients with MS and increasing cardiovascular disease occurrence. A review pointed out that most MS in males was slightly higher than that in females before age 50 and decreased after age 50 [60]. The LASSO regression results in this study showed that women were a risk factor for T2DM combined with MS, most of the enrolled females ranged from 48 to 65 years old, and most of them had entered perimenopause, which was related to the estradiol level mentioned above and similar to the results of similar studies [51–53].

Researchers around the world have developed risk prediction models for a variety of diseases and modes. In recent years, more and more risk prediction models for insulin resistance–related diseases such as T2DM and nonalcoholic fatty liver disease (NAFLD) have been developed based on Asian populations, especially East Asian populations [61–65]. However, these prediction models are limited studies on relatively low-risk individuals. Therefore, it is necessary to propose a new risk prediction model. We refer to the above studies and change the angle to develop a risk prediction model for T2DM patients with MS. The primary objective of current management of MS is to prevent the onset of clinical cardiovascular diseases and T2DM, while also aiming to prevent cardiovascular events in individuals with existing cardiovascular diseases. The CDS guidelines indicate that a scientifically and rationally designed therapeutic strategy for T2DM should be comprehensive, encompassing control of blood glucose, blood pressure, blood lipids, and body weight, with lifestyle modification as the foundation, supplemented by appropriate pharmacotherapy tailored to individual patient circumstances. In this study, the influencing factors of T2DM with MS selected by LASSO regression combined with multifactor logistic regression analysis are routine items that are easy to obtain clinically. We identified key features covering indicators related to blood glucose, blood pressure, blood lipids, and body weight. While verifying the constructed nomogram prediction model, it has good efficiency in prediction accuracy, calibration curve, and DCA, indicating that the prediction model has reasonable clinical practicability. This effectively assessed the congruence between the risk profile and local cardiovascular and T2DM screening guidelines. Healthcare providers can utilize this model to tailor screening and preventive measures according to the specific needs of patients, thereby reducing their risk of developing cardiovascular diseases or T2DM. However, obtaining all the laboratory indexes in the above model may not be possible at the grass-roots level and in the community due to the site and conditions. It is hoped that while vigorously developing primary medical care, indicators that impact various diseases can be popularized as soon as possible, and early screening and intervention can reduce the occurrence and development of related diseases in the future.

Although our nomogram performed well, there are some limitations to our study. First, this study is based on observation of hospitalized patients, and there may be unpredictable selection bias. Second, the study did not include the use of hypoglycemic, hypertensive, and lipid-lowering drugs, which may have a particular impact on the occurrence and development of MS. Third, this study is a cross-sectional study, unable to determine the causal relationship between each influencing factor and MS. Our study is a single-center data which can better reflect the situation of patients in Shenzhen. Although there are limitations, our prediction formula is also used for patients in Shenzhen. In future studies, we will expand the research center, expand the sample size, and carry out prospective cohort studies to verify more people, to further study this field. We will further improve the model in future studies by avoiding the use of components of MS as predictors to prevent potential tautological issues. To achieve this, we plan to introduce more independent predictors related to MS but not directly used as diagnostic criteria (e.g., inflammatory markers and insulin resistance indicators). Additionally, we will collaborate with multiple medical centers to expand the sample size and conduct multicenter validation to ensure the model's applicability across different populations. Furthermore, we will collect more data related to lifestyle and dietary factors (e.g., physical activity levels, dietary habits, and salt intake), which play significant roles in the development and progression of MS. By incorporating these factors, the model will be able to more comprehensively reflect the risk factors of MS and provide more precise guidance for clinical interventions.

## 5. Conclusion

The prediction model of this study covers four clinically easily obtained parameters: BMI, WC, TG, and HDL, and shows a high accuracy rate in the validation dataset. Our predictive model may provide an effective method for large-scale epidemiological studies of T2DM patients with MS and offer a practical tool for the early detection of MS in clinical work.

## Figures and Tables

**Table 1:** The baseline characteristics of T2DM patients with MS in the training and validation datasets.

	Training dataset		*p*	Validation dataset		*p*
	Non-MS group (*n* = 683)	MS group (*n* = 1431)		Non-MS group (*n* = 230)	MS group (*n* = 510)	
Age, years	57.00 (49.50–65.00)	56.00 (48.00–65.00)	0.027	57.00 (49.25–64.00)	57.00 (48.00–65.00)	0.896
Gender			< 0.001			0.257
Male	420 (61.49%)	1012 (70.72%)		150 (65.22%)	354 (69.41%)	
Female	263 (38.51%)	419 (29.28%)		80 (34.78%)	156 (30.59%)	
Smoking			< 0.001			0.006
No	455 (66.62%)	803 (56.11%)		154 (66.96%)	287 (56.27%)	
Yes	228 (33.38%)	628 (43.89%)		76 (33.04%)	223 (43.73%)	
Drinking			< 0.001			0.182
No	517 (75.70%)	981 (68.55%)		169 (73.48%)	350 (68.63%)	
Yes	166 (24.30%)	450 (31.45%)		61 (26.52%)	160 (31.37%)	
Hypertension			< 0.001			< 0.001
No	569 (83.31%)	585 (40.88%)		182 (79.13%)	211 (41.37%)	
Yes	114 (16.69%)	846 (59.12%)		48 (20.87%)	299 (58.63%)	
Coronary heart disease			< 0.001			0.329
No	630 (92.24%)	1251 (87.42%)		204 (88.70%)	439 (86.08%)	
Yes	53 (7.76%)	180 (12.58%)		26 (11.30%)	71 (13.92%)	
Diabetic complications			0.913			0.991
No	62 (9.08%)	132 (9.22%)		19 (8.26%)	42 (8.24%)	
Yes	621 (90.92%)	1299 (90.78%)		211 (91.74%)	468 (91.76%)	
Arteriosclerosis			0.496			0.252
No	155 (22.69%)	344 (24.04%)		66 (28.70%)	126 (24.71%)	
Yes	528 (77.31%)	1087 (75.96%)		164 (71.30%)	384 (75.29%)	
Course of diabetes			0.510			0.573
0–1 year	57 (8.35%)	142 (9.92%)		29 (12.61%)	49 (9.61%)	
1–5 years	134 (19.62%)	311 (21.73%)		43 (18.70%)	97 (19.02%)	
5–10 years	168 (24.60%)	337 (23.55%)		48 (20.87%)	116 (22.75%)	
10–20 years	241 (35.29%)	469 (32.77%)		71 (30.87%)	175 (34.31%)	
20–43 years	83 (12.15%)	172 (12.02%)		39 (16.96%)	73 (14.31%)	
BMI (kg/m^2^)	23.18 (21.30–25.18)	25.39 (23.50–27.68)	< 0.001	23.60 (21.50–25.89)	24.90 (23.20–27.39)	< 0.001
WC (cm)	86.00 (80.25–91.86)	93.00 (88.00–99.00)	< 0.001	86.00 (80.14–93.00)	93.00 (87.00–99.00)	< 0.001
Hip circumference (cm)	94.00 (90.00–98.25)	98.00 (94.00–103.00)	< 0.001	95.00 (90.05–98.00)	98.00 (94.00–102.20)	< 0.001
HbA1c (%)	8.17 (6.80–10.20)	8.70 (7.20–10.60)	< 0.001	8.00 (7.00–10.10)	8.25 (6.80–10.00)	0.981
TG (mmol/L)	1.11 (0.85–1.45)	1.96 (1.33–2.81)	< 0.001	1.05 (0.82–1.37)	1.83 (1.22–2.71)	< 0.001
TC (mmol/L)	4.20 (3.58–4.95)	4.23 (3.45–4.98)	0.486	4.22 (3.60–5.14)	4.43 (3.67–5.30)	0.131
LDL (mmol/L)	2.61 (2.00–3.24)	2.66 (2.02–3.29)	0.344	2.54 (1.91–3.29)	2.71 (2.03–3.41)	0.113
HDL (mmol/L)	1.21 (1.09–1.39)	0.98 (0.87–1.12)	< 0.001	1.17 (1.08–1.29)	0.91 (0.80–1.03)	< 0.001
UAL (mg/24h)	13.20 (7.99–30.54)	16.94 (9.60–44.28)	< 0.001	13.57 (7.96–29.60)	17.49 (9.01–43.80)	0.008
Cr (μmol/L)	62.10 (51.35–72.95)	65.90 (54.70–77.30)	< 0.001	64.65 (54.60–73.40)	69.35 (57.12–83.07)	< 0.001
UA (μmol/L)	330.00 (277.50–382.40)	367.10 (307.85–439.80)	< 0.001	323.45 (270.58–378.78)	361.85 (301.72–430.20)	< 0.001

*Note:* The differences in baseline characteristics between the non-MS and MS groups were assessed using Student's *t* test, the nonparametric Mann–Whitney *U* test for continuous variables and the *χ*^2^ Fisher's exact test for categorical variables.

**Table 2:** Prediction performance Model 1 and Model 2 for the risk of MS in T2DM.

Test	Model 1	Model 2
1	1431	1431
0	683	683
ROC area (AUC)	0.886	0.887
95% CI	0.871–0.901	0.872–0.902
Best threshold	0.758	0.764
Specificity	0.845	0.845
Sensitivity	0.806	0.809
Accuracy	0.819	0.821
Positive-LR	5.196	5.214
Negative-LR	0.229	0.226
Diagnose-OR	22.678	23.090
N-for-diagnose	1.536	1.529
Positive-pv	0.916	0.916
Negative-pv	0.676	0.679
a	1154	1158
b	106	106
c	277	273
d	577	577

**Table 3:** Multivariate analysis of influencing factors of MS in T2DM in the training dataset.

	Estimate	Std error	OR	95% CI	*p* value
(Intercept)	−9.182	0.882	0.000	0.000–0.001	< 0.001
BMI (kg/m^2^)	0.144	0.021	1.155	1.108–1.204	< 0.001
WC (cm)	0.092	0.008	1.097	1.080–1.113	< 0.001
TG (mmol/L)	1.058	0.091	2.880	2.409–3.442	< 0.001
HDL (mmol/L)	−3.300	0.280	0.037	0.021–0.064	< 0.001

**Table 4:** Prediction performance of the nomogram for the risk of MS in T2DM in the training and validation datasets.

Test	Training dataset	Validation dataset
1	1431	510
0	683	230
ROC area (AUC)	0.886	0.859
95% CI	0.871–0.901	0.831–0.887
Best threshold	0.758	1.223
Specificity	0.845	0.891
Sensitivity	0.806	0.667
Accuracy	0.819	0.737
Positive-LR	5.196	6.133
Negative-LR	0.229	0.374
Diagnose-OR	22.678	16.400
N-for-diagnose	1.536	1.792
Positive-pv	0.916	0.932
Negative-pv	0.676	0.547
a	1154	340
b	106	25
c	277	170
d	577	205

## Data Availability

The data supporting this study's finding are available from the author Jinghua Lai (ljh19901223@163.com) upon reasonable request.
